# An Extension of the Kimura Two-Parameter Model to the Natural Evolutionary Process

**DOI:** 10.1007/s00239-018-9885-1

**Published:** 2019-01-10

**Authors:** Takuma Nishimaki, Keiko Sato

**Affiliations:** Department of Information Sciences, Tokyo University of Science, Noda, Chiba 278-8510 Japan

**Keywords:** Evolutionary model, Genetic difference, K2P, Insertion, Deletion

## Abstract

Accurate estimates of genetic difference are required for research in evolutionary biology. Here we extend the Kimura two-parameter (K2P) model by considering gaps (insertions and/or deletions) and introduce a new measure for estimating genetic difference between two nucleotide sequences in terms of nucleotide changes that have occurred during the evolutionary process. Using the nuclear ribosomal DNA internal transcribed spacer 2 region from the genus *Physalis*, we demonstrate that species identification and phylogenetic studies strongly depend on evolutionary models. It is especially noteworthy that the use of different models affects the degree of overlap between intraspecific and interspecific genetic differences. We observe that the percentage of interspecific sequence pairs with values less than the maximum intraspecific genetic difference is 43.2% for the K2P model which is calculated by removing gap sites across all sequences, 22.7% for the K2P model which is calculated by removing gap sites for sequence pairs, and 16.9% for our model which is calculated without removing gap sites. Additionally, the numbers of sequence pairs with interspecific genetic differences of zero are 50 for the K2P model and 29 for our model. The genetic difference measure based on the K2P model, compared to our model, overestimates 21 sequence pairs that are not originally identical. These results indicate the importance of estimating genetic differences under the model of sequence evolution that includes insertions and deletions in addition to substitutions.

**Electronic supplementary material:**

The online version of this article (10.1007/s00239-018-9885-1) contains supplementary material, which is available to authorized users.

## Introduction

The Kimura two-parameter (K2P) model (Kimura 1980) is probably the most widely used of all models of nucleotide substitution for estimating genetic differences (generally called genetic distances) and phylogenetic relationships. It goes without saying that accurate models for evolution of molecular sequences are very important. However, the reason why the K2P model is overused in evolutionary studies and in DNA barcoding studies is not because the K2P model is the most precise model, but probably either because many authors have used it, or because it is the default of various packages for phylogenetic analyses.

DNA barcoding has been recognized as an efficient tool for species identification. Short DNA sequences from a standardized region of the genome are used as a DNA barcode to identify species. The DNA barcode of unknown specimen is compared with a reference library of DNA barcodes from known species by calculating pairwise genetic differences under a substitution model. The accuracy of DNA barcoding therefore depends on the choice of model. Misidentification of species is due to wide overlap between intra- and interspecific genetic differences (Luo et al. 2011; Meier et al. 2006; Meyer and Paulay 2005). Indeed, Barley and Thomson (2016) recently demonstrated that the use of different substitution models can have a substantial impact on the number of operational taxonomic units identified in barcoding data sets.

Nucleotide changes seen during the evolutionary process include substitutions, insertions, and deletions. The K2P model does not take into account the evolution by insertions and deletions. When estimating genetic difference using the K2P model for two aligned sequences, the sites with gaps (insertions and/or deletions) are removed. Although the K2P model is appropriate in some applications of nucleotide substitution, it is desirable for evolutionary models of molecular sequences to include insertions and deletions in addition to substitutions. So far, McGuire et al. (2001) have proposed an extension to a class of nucleotide substitution models to incorporate gap information. They treated a gap as a fifth character with the four nucleotides and demonstrated that it is better to incorporate gap information than to ignore it for phylogenetic inference. However, the transversion rate, insertion rate, and deletion rate in their model are all equal. We consider that this assumption is not suitable for evolutionary models because of different types of events.

In this paper, we extend the K2P model by assigning rates of insertions and deletions that differ from rates of substitutions and introduce a new measure for estimating genetic difference between two nucleotide sequences in terms of nucleotide changes that have occurred during the evolutionary process. Then, in order to evaluate the performance of our genetic difference measure, we investigate the accuracy of phylogenetic reconstruction for our difference measure and the K2P difference measure by using computer simulation. In addition, for the nuclear ribosomal DNA internal transcribed spacer 2 (ITS2) region from the genus *Physalis* which has been proposed as a universal DNA barcode to identify plants and animals (Yao et al. 2010), we calculate genetic differences using our difference measure and the K2P difference measure to compare these measures in the degree of overlap between intraspecific and interspecific genetic differences and in the inference of phylogenetic relationships. Finally, we discuss the importance of estimating genetic differences under the model of sequence evolution that includes insertions and deletions in addition to substitutions, for the development of evolutionary studies and DNA barcoding studies.

## Methods

### New Measure for Estimating Genetic Difference

Two sequences being compared are derived from a multiple alignment of homologous sequences, where $n$ is the length of the alignment. We focus on a pair of homologous sites in the two sequences and investigate how these sites are different from each other by nucleotide changes that have occurred during the evolutionary process extending over $t$ years since divergence from a common ancestor. We regard the sequence length in evolutionary process as being fixed. Note that the fixed length is $n$. Therefore, a deletion corresponds to the replacement of a nucleotide by a gap, and an insertion corresponds to the replacement of a gap by a nucleotide.

**Fig. 1:**
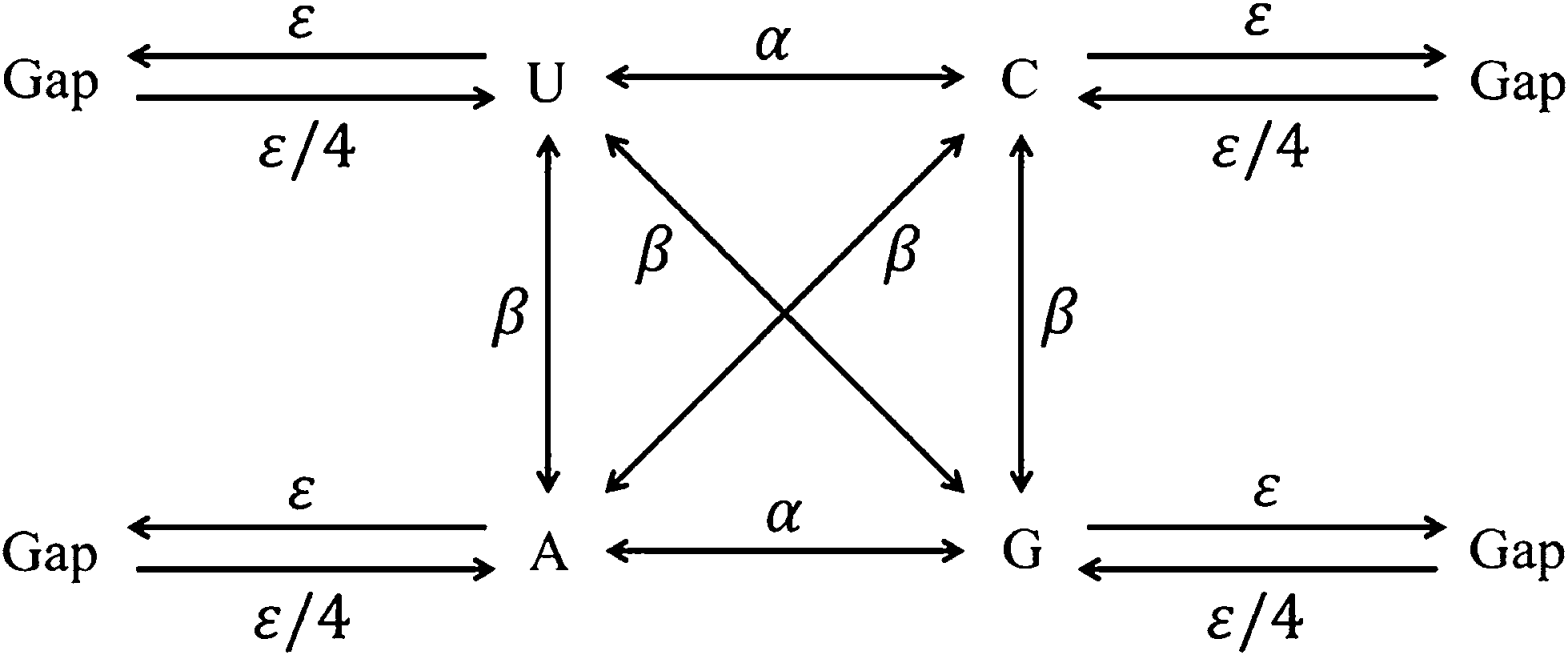
Evolutionary model of nucleotide changes and their rates per unit time

Here we assume an evolutionary model of nucleotide changes as shown in Fig. 1. The four nucleotides are denoted by A, C, G, and U in RNA. In case of DNA, we use the nucleotide T instead of U. Transitions and transversions occur at rate $\alpha$ and at rate $2\beta$ per site per unit time (year), respectively. In addition, deletions occur at rate $\varepsilon$ per site per unit time. On the other hand, assuming that a gap changes to any nucleotide with equal probability, the rate of change from a gap to a nucleotide is $\varepsilon /4$ when the total rate of insertions per site per unit time is $\varepsilon$. Therefore, the total rate of nucleotide changes per site per unit time $k$ is given by the following mixture:

1 $$k=w\left( {\alpha +2\beta +\varepsilon } \right)+\left( {1 - w} \right)\varepsilon,$$

where $w$ is the mixture weight, which means the probability that nucleotides exist in the two sequences. When we compare homologous sites in the two sequences, there are 25 combinations as shown in Table 1. We define three probabilities denoted by ${S_t}$, ${P_t}$, and ${Q_t}$, where ${S_t}$ is the probability of homologous sites showing identical nucleotides at $t$ years since divergence from a common ancestor, while ${P_t}$ and ${Q_t}$ are the probabilities of homologous sites showing nucleotide pairs of transition type and transversion type, respectively, at $t$ years. Moreover, we define two probabilities denoted by ${G_t}$ and ${N_t}$, where ${G_t}$ is the probability of homologous sites being occupied by pairs consisting of a nucleotide and a gap at $t$ years since the divergence, and ${N_t}$ is the probability of gap–gap at $t$ years. Note that $~{S_t}+{P_t}+{Q_t}+{G_t}+{N_t}=1$. Then, we can derive the following equations:

**Table 1:** Pairs of homologous sites in two sequences and the probability occupied by each pair at $t$ years since divergence from a common ancestor

Identical nucleotide pair	${\text{UU}}$	${\text{CC}}$	${\text{AA}}$	${\text{GG}}$	Total
Probability	${S_{1t}}$	${S_{2t}}$	${S_{3t}}$	${S_{4t}}$	${S_t}={S_{1t}}+{S_{2t}}+{S_{3t}}+{S_{4t}}$
Transition-type pair	${\text{UC}}$	${\text{CU}}$	${\text{AG}}$	${\text{GA}}$	Total
Probability	${P_{1t}}$	${P_{1t}}$	${P_{2t}}$	${P_{2t}}$	${P_t}=2{P_{1t}}+2{P_{2t}}$
Transversion-type pair	${\text{UA}}$	${\text{AU}}$	${\text{CG}}$	${\text{GC}}$	
Probability	${Q_{1t}}$	${Q_{1t}}$	${Q_{2t}}$	${Q_{2t}}$	
	${\text{UG}}$	${\text{GU}}$	${\text{AC}}$	${\text{CA}}$	Total
	${Q_{3t}}$	${Q_{3t}}$	${Q_{4t}}$	${Q_{4t}}$	${Q_t}=2({Q_{1t}}+{Q_{2t}}+{Q_{3t}}+{Q_{4t}})$
Nucleotide and gap pair	${\text{U}} -$	$- {\text{U}}$	${\text{C}} -$	$- {\text{C}}$	
Probability	${G_{1t}}$	${G_{1t}}$	${G_{2t}}$	${G_{2t}}$	
	${\text{A}} -$	$- {\text{A}}$	${\text{G}} -$	$- {\text{G}}$	Total
	${G_{3t}}$	${G_{3t}}$	${G_{4t}}$	${G_{4t}}$	${G_t}=2({G_{1t}}+{G_{2t}}+{G_{3t}}+{G_{4t}})$
Gap–gap pair	–				Total
Probability	${N_t}$				${N_t}$

2 $$\frac{{\Delta {S_t}}}{{\Delta t}} \equiv \frac{{{S_{t+\Delta t}} - {S_t}}}{{\Delta t}}= - 2\left( {\alpha +2\beta +\varepsilon } \right){S_t}+2\alpha ~{P_t}+2\beta ~{Q_t}+\frac{\varepsilon }{4}{G_t}~,$$

3 $$\frac{{\Delta {P_t}}}{{\Delta t}} \equiv \frac{{{P_{t+\Delta t}} - {P_t}}}{{\Delta t}}= - 2\left( {\alpha +2\beta +\varepsilon } \right){P_t}+2\alpha ~{S_t}+2\beta ~{Q_t}+\frac{\varepsilon }{4}{G_t}~,$$

4 $$\frac{{\Delta {Q_t}}}{{\Delta t}} \equiv \frac{{{Q_{t+\Delta t}} - {Q_t}}}{{\Delta t}}= - 2\left( {2\beta +\varepsilon } \right){Q_t}+4\beta ~{P_t}+4\beta ~{S_t}+\frac{\varepsilon }{2}{G_t}~,$$

5 $$\frac{{\Delta {N_t}}}{{\Delta t}} \equiv \frac{{{N_{t+\Delta t}} - {N_t}}}{{\Delta t}}= - 2\varepsilon {N_t}+\varepsilon {G_t}~,$$

where $\Delta t \ll 1$ stands for the length of a short time interval. Therefore, we can regard as $\Delta {S_t}/\Delta t \approx d{S_t}/dt$, $\Delta {P_t}/\Delta t \approx d{P_t}/dt$, $\Delta {Q_t}/\Delta t \approx d{Q_t}/dt$, $\Delta {N_t}/\Delta t \approx d{N_t}/dt$. Different nucleotide pairs do not exist at $t=0$, while matched pairs exist at $t=0,$ i.e., ${P_0}={Q_0}={G_0}=0$ and ${S_0}+{N_0}=1$. We consider that the probability of nucleotides in the ancestral sequence is equal to the probability of nucleotides in the two sequences. The following functions are the solutions of the differential equations with initial conditions ${P_0}={Q_0}=0$, ${S_0}={\text{~}}w{\text{~}}(0{\text{~}}<~w{\text{~}} \leqslant {\text{~}}1),$ and ${N_0}=1 - w$.

6 $${S_t}=\frac{1}{{16}}\left[ {1+{e^{ - 4\varepsilon t}} - 2{e^{ - 2\varepsilon t}}} \right]+\frac{w}{4}\left[ {{e^{ - 2\varepsilon t}}+2{e^{ - 2\left( {2\alpha +2\beta +\varepsilon } \right)t}}+{e^{ - 2\left( {4\beta +\varepsilon } \right)t}}} \right]~,$$

7 $${P_t}=\frac{1}{{16}}\left[ {1+{e^{ - 4\varepsilon t}} - 2{e^{ - 2\varepsilon t}}} \right]+\frac{w}{4}\left[ {{e^{ - 2\varepsilon t}} - 2{e^{ - 2\left( {2\alpha +2\beta +\varepsilon } \right)t}}+{e^{ - 2\left( {4\beta +\varepsilon } \right)t}}} \right]~,$$

8 $${Q_t}=~\frac{1}{8}\left[ {1+{e^{ - 4\varepsilon t}} - 2{e^{ - 2\varepsilon t}}} \right]+\frac{w}{2}\left[ {{e^{ - 2\varepsilon t}} - {e^{ - 2\left( {4\beta +\varepsilon } \right)t}}} \right]~,$$

9 $${N_t}=~\frac{1}{4}\left[ {1+{e^{ - 4\varepsilon t}}+2{e^{ - 2\varepsilon t}}} \right] - w{e^{ - 2\varepsilon t}}~.$$

By rearranging Eqs. (6–9), we obtain the following equations:

10 $$2{Q_t} - {N_t}= - {e^{ - 2\varepsilon t}} - w{e^{ - 2\left( {4\beta +\varepsilon } \right)t}}+2w{e^{ - 2\varepsilon t}}{\text{~}},$$

11 $$2{P_t} - {Q_t}=w{e^{ - 2\left( {4\beta +\varepsilon } \right)t}} - w{e^{ - 2\left( {2\alpha +2\beta +\varepsilon } \right)t}}~,$$

12 $${P_t} - {S_t}= - w{e^{ - 2\left( {2\alpha +2\beta +\varepsilon } \right)t}}~.$$

From Eqs. (10–12), we get

13 $$\alpha t=\frac{1}{8}{\text{log}}\frac{{w({P_t} - {Q_t}+{S_t})({P_t}+{Q_t}+{S_t} - {N_t})}}{{\left( {2w - 1} \right){{\left( {{S_t} - {P_t}} \right)}^2}}}~,$$

14 $$\beta t=\frac{1}{8}{\text{log}}\frac{{w({P_t}+{Q_t}+{S_t} - {N_t})}}{{\left( {2w - 1} \right)\left( {{P_t} - {Q_t}+{S_t}} \right)}}{\text{~}},$$

15 $$\varepsilon t=\frac{1}{2}{\text{log}}\frac{{2w - 1}}{{{P_t}+{Q_t}+{S_t} - {N_t}}}~.$$

Since the total rate of nucleotide changes including substitutions, insertions, and deletions per site per unit time is $k~=~w(\alpha +2\beta +\varepsilon )+\left( {1 - w} \right)\varepsilon$, the total number of nucleotide changes per site which separate the two sequences in the evolutionary process extending over $t$ years since divergence from a common ancestor is given by

16 $$K~=~2tk~=~2t\{ w(\alpha +2\beta +\varepsilon )+(1 - w)\varepsilon \} .$$

Then, substituting Eqs. (13–15) into Eq. (16) and omitting the subscript $t$ from ${S_t}$, ${P_t},$ and ${Q_t}$, we get

17 $$K~=~\frac{3}{4}w\log w - \frac{w}{2}\log \left( {S - P} \right)\sqrt {S+P - Q} .$$

This equation is useful as a measure for estimating genetic difference between two nucleotide sequences in terms of the number of nucleotide changes per site that have occurred in the evolutionary process extending over $t$ years. In this equation, $w$ is the probability that nucleotides exist in two sequences compared. $S={n_1}/n$, where ${n_1}$ is the number of sites that have identical nucleotides between the two sequences and $n$ is the total number of sites compared. $P={n_2}/n$, and $Q={n_3}/n$, where ${n_2}$ and ${n_3}$ are, respectively, the numbers of sites that have different nucleotides with respect to transition type and transversion type. Obviously, if gaps do not exist in two sequences compared (namely $w~=~1$), then Eq. (17) becomes equal to the equation for the K2P model.

### Simulation Analyses

In order to evaluate the performance of the difference measure in our model (K2P + Gap), we investigated the accuracy of phylogenetic reconstruction for both the K2P + Gap difference measure and the K2P difference measure by using computer simulation. Sequence data were simulated on perfect binary trees. For model trees of 16, 32, 64, 128, and 256 taxa, ancestral sequences of 250, 500, 750, and 1000 nucleotides in length were randomly generated under conditions of equal probability for each of the four nucleotides. Each ancestral sequence evolved along the perfect binary tree under ${P_{ij}}{\text{~}}$ = 0.001 (low), 0.005 (medium), and 0.01 (high) per site per branch, where${\text{~}}{P_{ij}}({\text{~}}i,j \in {\text{~}}\{ {\text{A}},{\text{C}},{\text{G}},{\text{T}},{\text{or~Gap}}\} )$ is the probability from *i* to *j* ($\ne$*i*). In total, we had 60 model conditions (five numbers of taxa, four sequence lengths, and three change rates). 100 replicates were performed for each model condition. The sequence data obtained at the leaf node were given as input to the phylogenetic reconstruction. For each data set, the K2P genetic difference matrix and our genetic difference matrix were calculated to reconstruct phylogenetic trees, using neighbor-joining method (Saitou and Nei 1987). The genetic differences of K2P were calculated after removal of gap sites across all the sequences (complete deletion) and also after removal of gap sites for the sequence pairs (pairwise deletion). On the other hand, the genetic differences of K2P + Gap were calculated without eliminating gaps. The accuracy of phylogenetic reconstruction was evaluated as the percentage of replications in which the correct topology was obtained when compared to the model tree.

### Genetic Data Analyses

We additionally used 86 ITS2 sequences of 45 species from the genus *Physalis* described by Feng et al. (2016) to compare the performance of the K2P + Gap difference measure with the K2P difference measure. Multiple alignment of the ITS2 sequences were performed with ClustalW2 with default parameters (Larkin et al. 2007), and then each genetic difference was calculated for a total of 3,655 sequence pairs of 45 *Physalis* species listed in Table 2. The total aligned sequence length was 225 nucleotides. The genetic differences of K2P were calculated with both complete deletion of gaps and pairwise deletion of gaps. On the other hand, the genetic differences of K2P + Gap were calculated without eliminating gaps.

**Table 2:** 45 *Physalis* species used in this study

Species name	No. of sequence	Species name	No. of sequence
*P. angulate*	7	*P. hederaefolia var. puberula*	1
*P. angulatta var. villosa*	4	*P. heterophylla*	1
*P. acutifolia*	1	*P. lanceolata*	1
*P. crassifolia*	2	*P. longifolia*	2
*P. lagascae*	1	*P. peruviana*	2
*P. microcarpa*	1	*P. pumila*	1
*P. philadelphica*	1	*P. sordida*	1
*P. campanulata*	1	*P. virginiana*	2
*P. glutinosa*	1	*P. minimaculata*	2
*P. carpenteri*	2	*P. angustifolia*	1
*P. chenipodifolia*	1	*P. cinerascens*	2
*P. coztomatl*	2	*P. mollis*	1
*P. greenmanii*	1	*P. viscosa*	1
*P. hintonii*	2	*P. minima*	6
*P. pubescens*	9	*P. lassa*	1
*P. angustiphysa*	1	*P. arenicola*	2
*P. cordata*	1	*P. alkekengi var. franchetii*	7
*P. pruinosa*	1	*P. alkekengi*	3
*P. ignota*	1	*P. arborescens*	2
*P. nicandroides*	1	*P. melanocystis*	1
*P. patula*	1	*P. walteri*	1
*P. caudella*	1	*P. microphysa*	1
*P. hederaefolia*	1		

The intraspecific genetic differences between all sequences collected within each species and the interspecific genetic differences between all species in the genus *Physalis* were calculated to examine the degree of overlap between intra- and interspecific genetic differences. The mean of the interspecific differences was calculated for a total of 113 sequence pairs from 17 species with at least two sequences. The mean of the interspecific differences was calculated for a total of 3,542 sequence pairs. The degree of overlap was calculated as the percentage of interspecific sequence pairs with values less than the maximum intraspecific difference (The number of interspecific sequence pairs in the overlap zone divided by the total number of interspecific sequence pairs × 100).

To further examine how different evolutionary models affect the phylogenetic relationships among species from the genus *Physalis*, phylogenetic trees were generated by the neighbor-joining method with our model and with the K2P model.

## Results

### Accuracy of Phylogenetic Reconstruction

K2P + Gap had the best accuracy for any of all 60 model conditions (Supplementary Fig. S1). Table 3 shows a summary of the simulation results. The accuracy of phylogenetic reconstruction decreases as the number of taxa increases. This was particularly notable for K2P with complete deletion. In the case of K2P with complete deletion, in comparison to others, the accuracy was extremely low for the three rates of change (low, medium, and high). On the other hand, in the case of K2P with pairwise deletion, the accuracy was much higher than that of K2P with complete deletion for any conditions. Above all, as seen in Table 3, K2P + Gap shows the highest accuracy of the three measures.

**Table 3:** Percentage of replications in which the correct topology was obtained

Change rate	Number of taxa	K2P (complete deletion) (%)	K2P (pairwise deletion) (%)	K2P + Gap (%)
Low (0.001 per site per branch)	16	39.3	41.3	53.8
	32	21.3	23.8	39.8
	64	3.8	8.5	21.3
	128	0.3	1.5	8.0
	256	0.0	0.3	1.8
Medium (0.005 per site per branch)	16	92.8	94.3	96.0
	32	79.0	82.8	90.8
	64	48.5	72.3	83.0
	128	2.8	60.0	72.8
	256	0.0	39.3	58.8
High (0.01 per site per branch)	16	96.8	96.8	98.5
	32	78.8	89.5	93.5
	64	29.5	80.0	89.5
	128	0.0	66.0	78.0
	256	0.0	53.3	68.3

Each percentage was averaged across 250, 500, 750, and 1000 nucleotides in length

### Effect of Model Selection on DNA Barcoding and Phylogenetic Studies

**Fig. 2:**
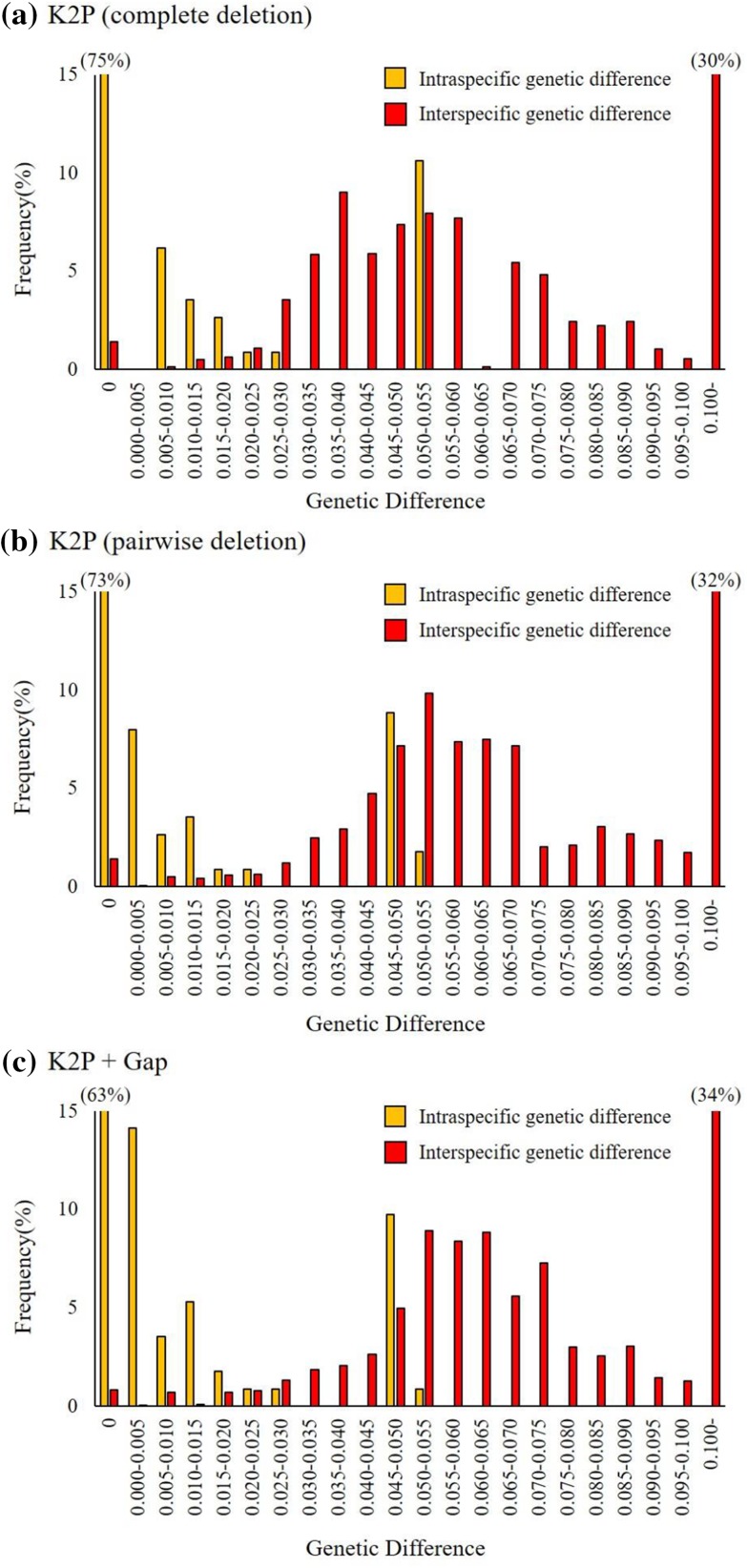
Frequency distribution of intra- and interspecific genetic differences in 86 ITS2 sequences of 45 species from the genus *Physalis*. Genetic differences were calculated for 113 intraspecific sequence pairs and 3542 interspecific sequence pairs using (**a**) K2P difference measure with complete deletion of gaps, (**b**) K2P difference measure with pairwise deletion of gaps, and (**c**) K2P + Gap difference measure

Genetic differences of 86 ITS2 sequences of 45 species from the genus *Physalis* were calculated under both our model and the K2P model. We examined their respective intra- and interspecific relationships to compare and evaluate the performance of the different measures (Fig. 2). The intraspecific genetic differences ranged from 0 to 0.0544 for K2P with complete deletion, from 0 to 0.0508 for K2P with pairwise deletion, and from 0 to 0.0503 for K2P + Gap. 75.2%, 73.5%, and 62.8% of the sequence pairs with intraspecific differences were zero for K2P with complete deletion, K2P with pairwise deletion, and K2P + Gap, respectively. Meanwhile, the interspecific genetic differences ranged from 0 to 0.1703 for K2P with complete deletion, from 0 to 0.1651 for K2P with pairwise deletion, and from 0 to 0.1662 for K2P + Gap. For K2P + Gap, the sequence pairs with interspecific differences of zero were 0.8% (29 sequence pairs). These sequence pairs were completely identical. For K2P with complete deletion and K2P with pairwise deletion, the sequence pairs with interspecific differences of zero were both 1.4% (50 sequence pairs). The mean intraspecific and interspecific differences, and the degree of overlap between intraspecific and interspecific genetic differences are given in Table 4. The percentage (number) of interspecific sequence pairs with values less than the maximum intraspecific difference was 43.2% (1531) for K2P with complete deletion, 22.7% (804) for K2P with pairwise deletion, and 16.9% (600) for K2P + Gap. When the highest 5% of the intraspecific differences and the lowest 5% of the interspecific differences were excluded, the degree of overlap was 38.2%, 16.9%, and 8.5%, respectively. The overlap in K2P + Gap was extremely small in comparison with others.

**Table 4:** Analyses of intra- and interspecific genetic differences in 86 ITS2 sequences of 45 species from the genus *Physalis*

	Mean intraspecific difference	Mean interspecific difference	Overlap (%)	No. of species pairs (sequence pairs) with interspecific differences of zero
K2P (complete deletion)	0.007 ± 0.017	0.073 ± 0.038	43.2	4 (50)
K2P (pairwise deletion)	0.007 ± 0.015	0.079 ± 0.035	22.7	4 (50)
K2P + Gap	0.007 ± 0.015	0.082 ± 0.037	16.9	2 (29)

**Fig. 3:**
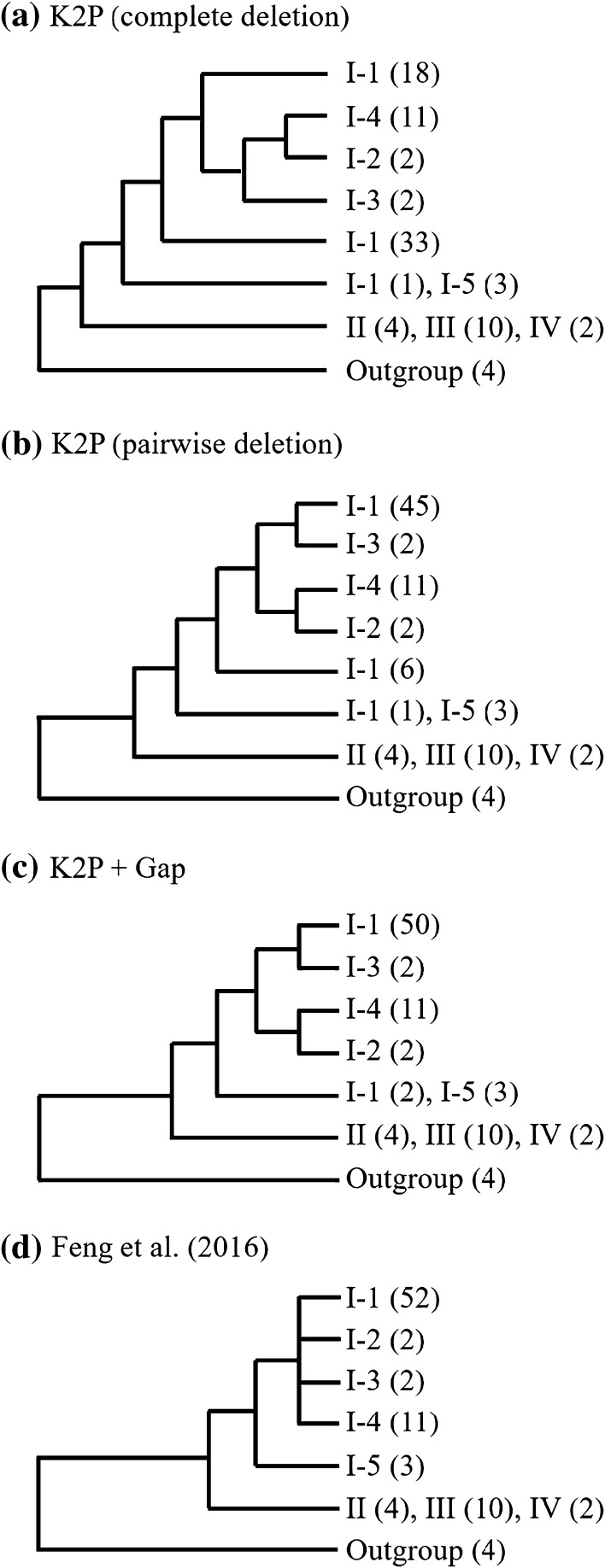
Simplified phylogenetic trees among *Physalis* with (**a**) the NJ method based on K2P difference measure with complete deletion of gaps, (**b**) the NJ method based on K2P difference measure with pairwise deletion of gaps, (**c**) the NJ method based on K2P + Gap difference measure, and (**d**) the ML method obtained by Feng et al. (2016). The number in parentheses is the number of the sequences

We additionally constructed phylogenetic trees by the neighbor-joining (NJ) method using the above genetic differences (Supplementary Fig. S2). The results by K2P with complete deletion, K2P with pairwise deletion, and K2P + Gap gave different phylogenetic topologies. In accordance with the four clusters I, II, III, and IV on the phylogenetic tree with the maximum likelihood (ML) method provided by Feng et al. (2016), the relationships among the species of the genus *Physalis* are shown in the simplified phylogenetic trees of Fig. 3. All NJ topologies differed from the ML topology. Subcluster I-1 containing 52 sequences were divided into three lineages in both the NJ trees based on K2P with complete deletion and pairwise deletion. In the NJ tree based on K2P + Gap, subcluster I-1 were divided into two lineages, where part of subcluster I-1 containing two sequences were merged into subcluster I-5 as shown in Fig. 3c, because the two sequences of subcluster I-1 were all far away from other sequences of subcluster I-1. Overall, the phylogenetic classification of *Physalis* with the NJ method based on K2P + Gap was congruent with that with the ML method.

## Discussion

Sequence alignment and estimation of genetic difference are crucial steps in molecular evolutionary studies and DNA barcoding studies. Recent advances in alignment algorithms (e.g., Edgar 2004; Hara et al. 2010; Katoh and Standley 2013; Larkin et al. 2007; Sievers et al. 2011) lead to the determination of the correct location of insertions and deletions that have occurred in either of the two sequences since their divergence from a common ancestor. Therefore, with the improvement in accuracy of sequence alignment, it is necessary to incorporate the evolutionary information of sites containing gaps into measures for estimating genetic differences.

In this study, we extended the K2P model by considering gaps and introduced a measure for estimating genetic difference between two nucleotide sequences in terms of nucleotide changes that have occurred during the evolutionary process. Our simulation results indicated that the accuracy of using our model is consistently better than those using the K2P model. Furthermore, as for the ITS2 sequences of *Physalis* species, we observed a large overlap between intra- and interspecific genetic differences for the K2P model (K2P with complete deletion, 43.2%; K2P with pairwise deletion, 22.7%), and a relatively small overlap for our model (K2P + Gap, 16.9%). In addition, the sequence pairs with interspecific genetic differences of zero were 50 sequence pairs for K2P and 29 sequence pairs for K2P + Gap. This means that how sequences with homologous sites consisting of a nucleotide and a gap have been treated as completely identical sequences. It is obvious that removal of gap sites and evolutionary models which ignore gaps cause misidentification and misclassification of species. Also, the phylogenetic comparison based on the ITS2 sequences showed phylogenetic inference relies on evolutionary models. Clearly, it is desirable to use the most appropriate and informative measure for accurate estimates of genetic difference. We believe that appropriately incorporating the evolutionary information of sites containing insertions and deletions into genetic difference measures for not only the K2P model but also other evolutionary models will be helpful to detect meaningful difference in an evolutionary process and facilitate accurate species identification and classification.

## Electronic supplementary material

Below is the link to the electronic supplementary material.

Supplementary material 1 (PDF 353 KB)

Supplementary material 2 (PDF 935 KB)
