# Supplementary material for: An Extension of the Kimura Two-Parameter Model to the Natural Evolutionary Process
Source: J Mol Evol. 2019 Jan 10;87(1):60–7. doi: 10.1007/s00239-018-9885-1 (PMC6514111; doi:10.1007/s00239-018-9885-1)
Supplement: Supplementary file 1 — Supplementary material 1 (PDF 353 KB) [file 239_2018_9885_MOESM1_ESM.pdf]

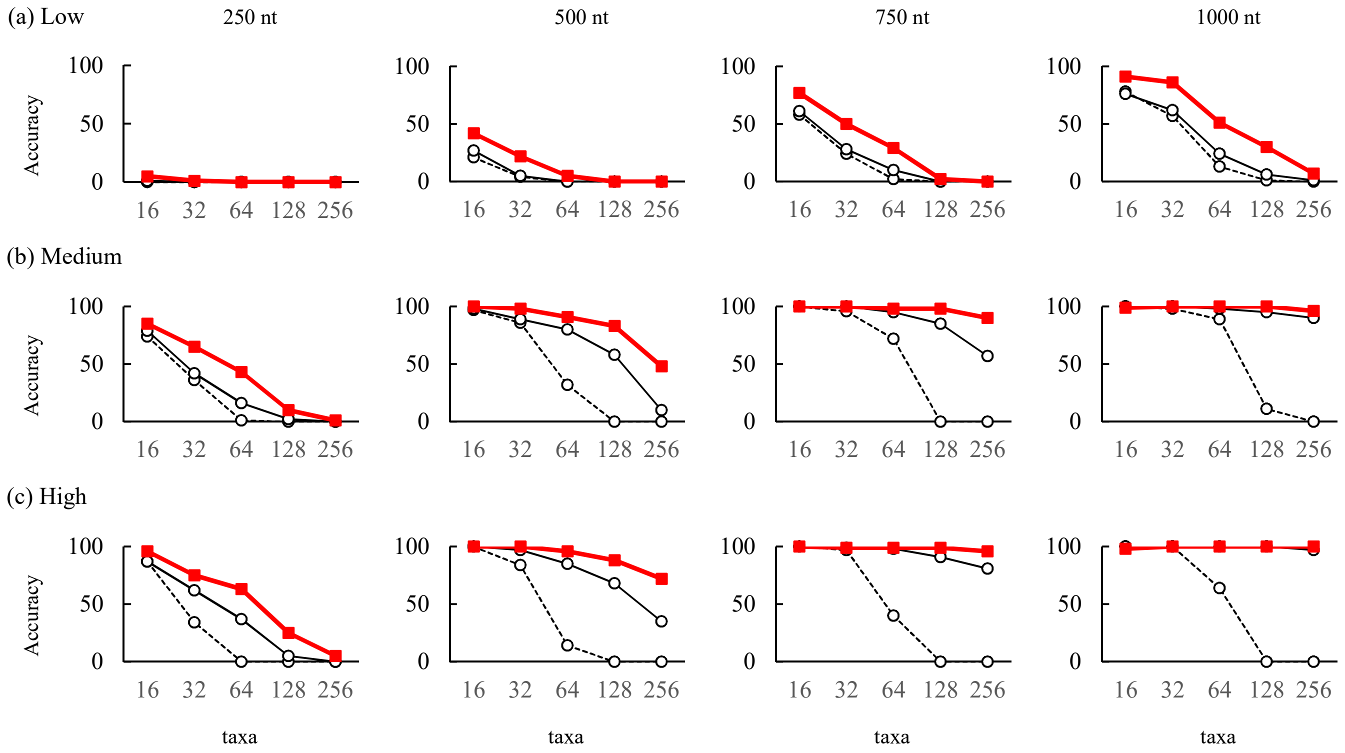

Fig. S1. Comparison of accuracy of phylogenetic reconstruction for K2P + Gap (*red line*) versus K2P with pairwise deletion (*solid line*) and K2P with complete deletion (*dashed line*). Data sets were simulated under varying numbers of taxa, sequence lengths, and change rates: (a) 0.001 (Low), (b) 0.005 (Medium), and (c) 0.01 (High) per site per branch
