# Supplementary material for: An Extension of the Kimura Two-Parameter Model to the Natural Evolutionary Process
Source: J Mol Evol. 2019 Jan 10;87(1):60–7. doi: 10.1007/s00239-018-9885-1 (PMC6514111; doi:10.1007/s00239-018-9885-1)
Supplement: Supplementary file 2 — Supplementary material 2 (PDF 935 KB) [file 239_2018_9885_MOESM2_ESM.pdf]

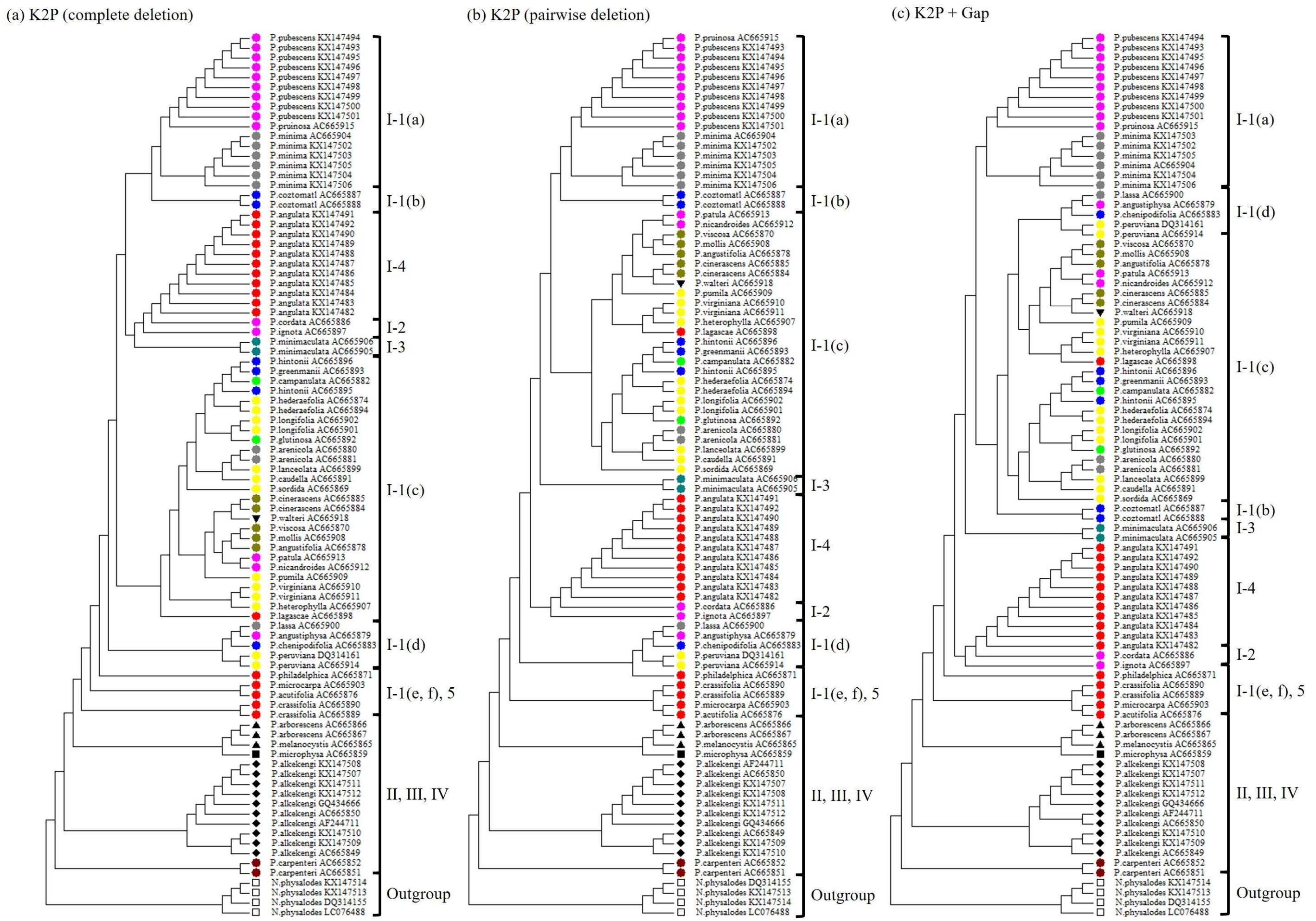

Fig. S2. Phylogenetic trees based on 86 ITS2 sequences of 45 species from the genus *Physalis* and 4 ITS2 sequences from the genus *Nicandra* (outgroup). Phylogenetic trees were constructed by the NJ method based on genetic differences estimated using (a) K2P difference measure with complete deletion, (b) K2P difference measure with pairwise deletion, and (c) K2P + Gap difference measure. The classification by color and cluster designation are in accordance with those on the ML tree provided by Feng et al. (2016)
